# Ionizing radiation induces endothelial transdifferentiation of glioblastoma stem-like cells through the Tie2 signaling pathway

**DOI:** 10.1038/s41419-019-2055-6

**Published:** 2019-10-28

**Authors:** Pauline Deshors, Christine Toulas, Florent Arnauduc, Laure Malric, Aurore Siegfried, Yvan Nicaise, Anthony Lemarié, Dorian Larrieu, Marie Tosolini, Elizabeth Cohen-Jonathan Moyal, Monique Courtade-Saidi, Solène M. Evrard

**Affiliations:** 1 INSERM UMR 1037, Toulouse Cancer Research Center, Toulouse, France; 2 Institut Claudius Regaud, IUCT-O, Toulouse, F-31059 France; 3 University of Toulouse III Paul Sabatier, Toulouse, France; 4 Pathology and Cytology Department, Toulouse CHU, IUCT Oncopole, Toulouse, France; 5 Faculty of Pharmaceutical Sciences, University of Toulouse III Paul Sabatier, Toulouse, France

**Keywords:** Cancer stem cells, Radiotherapy, CNS cancer

## Abstract

Glioblastomas (GBM) are brain tumors with a poor prognosis despite treatment that combines surgical resection and radio-chemotherapy. These tumors are characterized by abundant vascularization and significant cellular heterogeneity including GBM stem-like cells (GSC) which contribute to tumor aggressiveness, resistance, and recurrence. Recent data has demonstrated that GSC are directly involved in the formation of new vessels via their transdifferentiation into Tumor Derived Endothelial Cells (TDEC). We postulate that cellular stress such as ionizing radiation (IR) could enhance the transdifferentiation of GSC into TDEC. GSC neurospheres isolated from 3 different patients were irradiated or not and were then transdifferentiated into TDEC. In fact, TDEC obtained from irradiated GSC (TDEC IR+) migrate more towards VEGF, form more pseudotubes in Matrigel^TM^ in vitro and develop more functional blood vessels in Matrigel^TM^ plugs implanted in Nude mice than TDEC obtained from non-irradiated GSC. Transcriptomic analysis allows us to highlight an overexpression of Tie2 in TDEC IR+. All IR-induced effects on TDEC were abolished by using a Tie2 kinase inhibitor, which confirms the role of the Tie2 signaling pathway in this process. Finally, by analyzing Tie2 expression in patient GBMs by immunohistochemistry, we demonstrated that the number of Tie2+ vessels increases in recurrent GBM compared with matched untreated tumors. In conclusion, we demonstrate that IR potentiates proangiogenic features of TDEC through the Tie2 signaling pathway, which indicates a new pathway of treatment-induced tumor adaptation. New therapeutic strategies that associate standard treatment and a Tie2 signaling pathway inhibitor should be considered for future trials.

## Introduction

The primitive tumors of the central nervous system represent ~2–3% of all human cancers. The multiform glioblastoma (GBM), classified as a grade IV astrocytoma according to the classification of the World Health Organization, is the most aggressive and the most frequent of these tumors in adults^1^. Despite treatments that combine radiotherapy and chemotherapy, average survival still remains between 12 and 15 months^2–4^. One of the main clinical characteristics of these tumors is their recurrence even within the fields of ionizing radiation (IR). The presence of chemo/radioresistant tumor cells could be responsible for these aggressive recurrences. In fact, it is now established that GBM are very heterogeneous tumors, as are most solid cancers^5^. Recent studies highlight the presence within the tumor of a subpopulation of self-renewing and pluripotent GBM stem-like cells (GSC), also called GBM-initiating cells. These GSC are characterized by their ability to self-renew in vitro (neurosphere formation) and in vivo, by a high expression of neural stem cell markers (i.e. Olig2, Nestin) and stem cell transcription factors (i.e. Sox2, Nanog), their pluripotent aptitude to differentiate into neurons, astrocytes or oligodendrocytes and their high tumorigenic potential in vivo in orthotopically xenografted mice^6,7^. In addition, the presence of these GSC could explain the high rate of GBM recurrence, since they were shown to be extremely tumorigenic and radioresistant^8^.

In addition, GBMs are characterized by significant vascularization. The formation of vessels connected to the tumor develops prematurely during tumor progression. This vascularization, although abundant, is morphologically and functionally abnormal and therefore, contributes to the establishment of hypoxia within the tumor, which helps in GSC stemness maintenance. Moreover, GSC are mostly found near vessels within niches formed by endothelial cells^9^. It has been shown that endothelial cells and GSC establish connections, which are important for GSC stemness maintenance^9^. Various mechanisms of glioma-associated neovascularization have been described, such as vascular co-option, angiogenesis, vasculogenesis and vascular mimicry^10^. Furthermore, several teams have shown the existence of cells with endothelial characteristics called Tumor Derived Endothelial Cells (TDEC), which come from the transdifferentiation of GBM cells and more particularly from GSC^11–13^. When GSC were cultured in endothelial media for a few days, they generated cells with phenotypic and functional characteristics of endothelial cells. Moreover, the vessels of tumor xenografts formed by the injection of GSC in immunocompromised mice were primarily composed of human endothelial cells^11^. This new mechanism of glioma-associated neovascularization could contribute to the failure of the anti-angiogenic therapies (anti-VEGF), which is mainly associated with enhanced invasiveness^14^.

Considering the importance of vascularization in GBM and in the stemness maintenance of GSC, the capacity of GSC for glio-endothelial transitioning could then participate in the recurrence of GBM after treatment, especially after radiotherapy. In fact, it was shown that IR enhances tumor revascularization^15,16^. Although the authors showed that the mechanism involved was vasculogenesis, it cannot be excluded that another mechanism might be implicated, such as the transdifferentiation of radioresistant GSC into endothelial cells. Quite recently, our team demonstrated that subtoxic IR exposure of GBM differentiated cells (GDC) isolated from patient tumor resections potentiates the long-term reacquisition of stem-associated properties. These include the ability to generate primary and secondary neurospheres, the expression of stemness markers and an increase in tumorigenicity, which suggest that GBM cells display significant plasticity behavior after cellular stress such as IR^17^.

Because of the impact of IR on tumor revascularization and on GBM cell plasticity, we hypothesized that IR may impact GSC transdifferentiation into TDEC. Through our work, it is shown for the first time that radiotherapy can promote proangiogenic features of TDEC in GBM in vitro and in vivo, which may participate in tumor revascularization after treatment and finally contribute to in field recurrence of these aggressive cancers.

## Materials and methods

### Human tumor collection

The study was conducted on newly diagnosed GBM samples isolated from 3 different patients to establish 3 primary GSC cell lines (SRA5, SRB1 and SRC3). All samples were obtained as part of the STEMRI clinical trial (NCT01872221) with written informed consent from patients admitted to the Neurosurgery Department at Toulouse University Hospital and were processed in accordance with the Institution’s Human Research Ethics Committee. Tumors used in this study were histologically diagnosed as grade IV astrocytoma according to the WHO criteria.

### Cell culture and treatments

The GBM samples were processed as described previously^17–20^ in order to obtain primary neurospheres shown to be enriched in GSC. Neurosphere GSC were maintained in Stem Cell Medium (SCM) at 37 °C in 5% CO_2_ and were fully characterized in previous works^17–20^. These cells were used between the 2nd and 10th passages in order to avoid any stem cell property loss. The stem cell medium was composed of DMEM-F12 (Lonza) supplemented with B27 and N2 (Invitrogen), 25 ng/ml of FGF-2 and EGF (Peprotech).

HUVEC (Human Umbilical Vein Endothelial Cells, Lonza) were cultured in EGM-2 medium (Endothelial Growth Medium, Lonza).

**Fig. 1:**
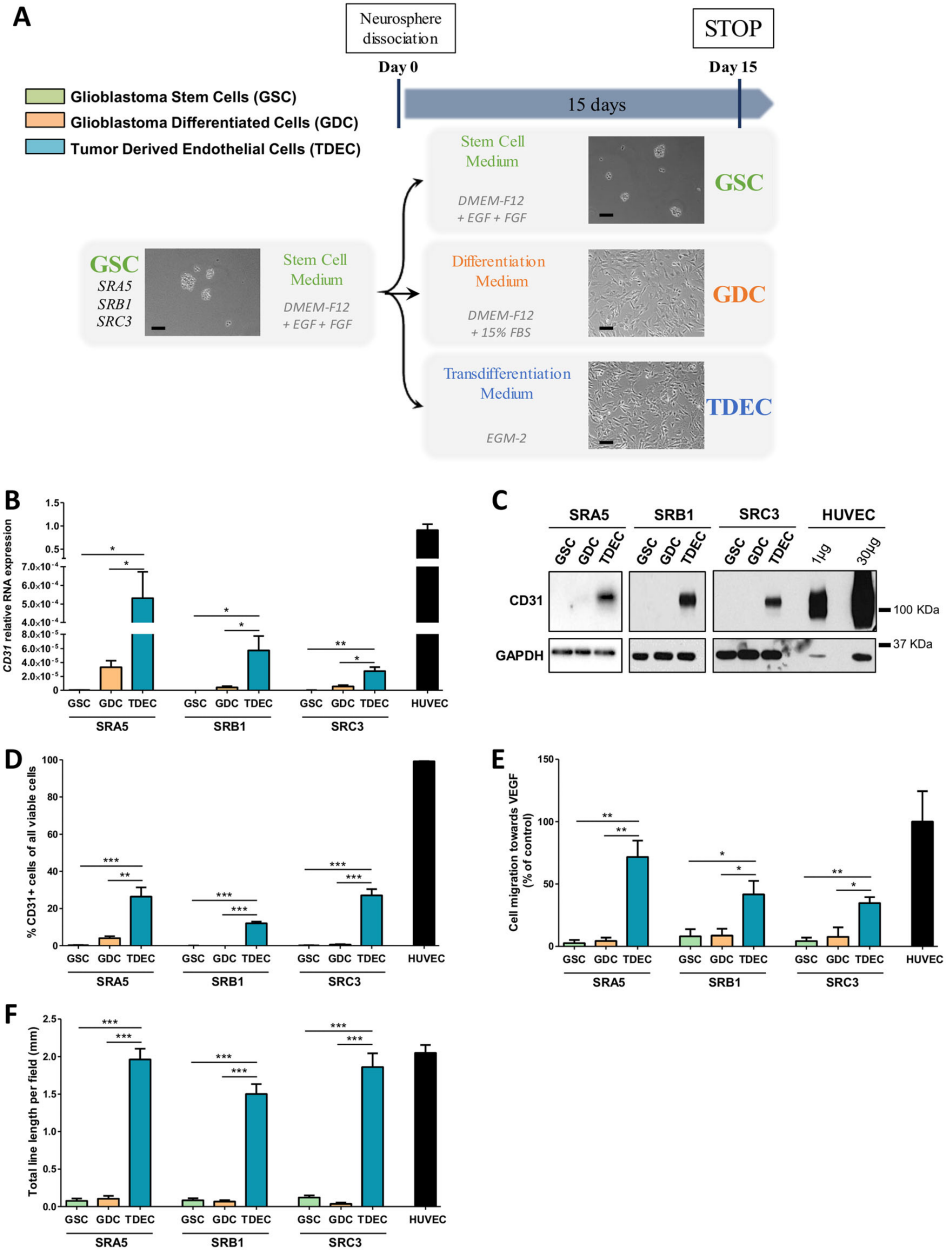
GSC cultured under conditions of endothelial differentiation develop phenotypical and functional features of endothelial cells. **a** Overview of the different cell culture protocols. As described in the Materials and Methods section, GSC-enriched neurospheres were isolated from patient samples and cultured in a specific stem cell medium (DMEM-F12 with EGF (*epidermal growth factor)* and FGF (*fibroblast growth factor)* growth factors). Neurospheres were then dissociated and placed for at least 15 days (i) in stem cell medium to keep GSC in culture as a control, (ii) in differentiation medium (DMEM-F12 with 15% FBS (*fetal bovine serum)* to obtain GDC or (iii) in transdifferentiation medium (EGM-2) to obtain TDEC. Scale bars, 100 µm. **b** Relative RNA expression of the endothelial marker CD31 determined by RT-qPCR in GSC, GDC, TDEC and HUVEC. Results are normalized to HUVEC expression. **c** Immunoblot of CD31 in GSC, GDC, TDEC, and HUVEC. Blots are representative of at least 3 independent experiments in the three patients’ GSC lines (SRA5, SRB1, and SRC3). **d** FACS immunofluorescence analysis of CD31 protein expression in GSC, GDC, TDEC and HUVEC. The graph represents means ± SEM of the percentage of CD31 positive cells among all viable cells of at least 3 independent experiments. **e** Percentage of cells that migrate towards VEGF normalized to HUVEC. **f**. Pseudotube formation assay. The graph represents means ± SEM of the total line length per field determined by the quantification of at least 3 fields per well

Differentiation was performed according to a previously published protocol adapted as follows^17^. Briefly, GSC neurospheres were dissociated and then cells were cultured and plated as an adherent monolayer (at least 4 × 10^3^ cells/cm²) in DMEM-F12 supplemented with 15% FCS (Sigma) (Differentiation Medium) for 15 days to ensure optimum differentiation and thereby obtain GDC (Fig. 1a). For transdifferentiation, GSC neurospheres were dissociated and then cells were cultured and plated as an adherent monolayer (at least 4 × 10^3^ cells/cm^2^) in EGM-2 medium (transdifferentiation medium) for 15 days (Fig. 1a).

After 15 days of culture, the totality of the cells was collected after trypsinization for subsequent experiments.

For Tie2 inhibition analysis, cells were treated with 2 µM of a Tie2 kinase inhibitor (Tie2i) (Abcam) diluted in DMSO for 15 days of transdifferentiation^21^.

### Irradiation

Dissociated GSC were maintained in stem cell medium for 6 h and subjected to a 2, 3 or 2 × 2 Gy IR with a GammaCell Exactor 40 (Nordion). After IR, GSC were kept in stem cell medium for 24 h. Cells were then placed either in (i) differentiation medium, (ii) transdifferentiation medium or (iii) kept in stem cell medium for 15 days. As no significant differences were observed between the different doses of IR tested (data not shown), we only used doses of 2Gy in this paper, which is equivalent to the daily dose used for GBM patients.

### Cell proliferation analysis

Cells were plated in 96-well plates at a density of 5 × 10^3^ cells per well and were incubated at 37 °C in 5% CO_2_ for 24 h. The proliferation ability was assessed by using WST-1 reagent (Roche) and all samples were run in triplicate. WST-1 reagent was added to the wells and cells were incubated for 2 h at 37 °C in 5% CO_2_. The absorbance was determined with a microplate reader (FLUOstar OPTIMA) at a wavelength of 540 nm.

### Quantitative real-time RT-PCR

Total RNA was isolated either from HUVEC, GSC, differentiated or transdifferentiated cells using the RNeasy Plus Mini kit (Qiagen) and then reverse-transcribed using the iScript^TM^ cDNA synthesis kit (Bio-Rad). Real-time qPCR reactions were tested using SsoFAST^TM^ EvaGreen® Supermix dye (Biorad) and ABI-StepOnePlus Detection System (Applied Biosystems). 18S rRNA (18S) was used as an endogenous control in the ΔCt analysis. The different primers (Eurogentec) used in this study are described in Supplementary Table S1.

### Western blotting

Cells were lysed in RIPA buffer complemented with protease and phosphatase inhibitor cocktails (Sigma). Protein content was quantified using Bradford Reagent (Biorad) and 30 µg of protein were then separated on a 7.5 or 10% SDS–PAGE, electroblotted onto PVDF membranes (Amersham). Membranes were then blocked with 10% milk for 1 h. Primary antibodies used for this study are listed in Supplementary Table S2. Primary antibodies were incubated overnight and then the membranes were washed. After incubation with HRP-linked secondary antibodies (anti-mouse (Abcam, 1/10 000), anti-rabbit (Abcam, 1/10 000), the reaction was developed with Western ECL-substrate (Thermo Scientific).

### Flow cytometry analyses

For all samples, 2 × 10^5^ cells were first incubated for 30 min in PBS with 10% FCS at 4 °C to avoid nonspecific binding and then incubated with appropriate conjugated primary antibodies for 1 h at 4 °C in the dark. Fluorescence related to immunolabeling was measured using an Accuri C6 flow cytometer (BD Biosciences) and a total of at least 10,000 events were recorded for each sample. The antibodies that were used are indicated in Supplementary Table S2.

### ELISA assay

Cell culture supernatants were harvested and centrifuged to remove cells and debris. Human Angiopoietin-1 (ANG1) and Angiopoietin-2 (ANG2) ELISA (R&D Systems) were performed according the manufacturer’s instructions. All samples were analysed in duplicate and analyzed with a plate reader (FLUOstar OPTIMA). Readings at 540 nm were subtracted from readings at 450 nm and growth factor amounts were determined using a standard curve.

### AcLDL uptake assay

For the AcLDL uptake assay, 10 µg/ml of diI-acLDL (1,1′-dioctadecyl-3,3,3′,3′-tetramethylindocarbocyanine perchlorate - acLDL) (Molecular Probes, Life Technologies) were added to culture medium. The cells were then cultured at 37 °C in 5% CO_2_ for 4 h [12, 22]. Cells were then washed and fixed in Paraformaldehyde 4% (ChemCruz). After washing, cells were stained with PBS containing 0.1% DAPI for 10 min and fluorescence related to acLDL uptake in cells was observed and photographed (Nikon NIS Element).

### Pseudotube formation assay

For pseudotube formation assay, 80 µl of Growth Factor Reduced Matrigel^TM^ (Corning) were dropped into wells of a 96-wells plate and allowed to polymerize for 1 h at 37 °C. Cells were then seeded at 15 × 10^3^ cells per well in a complete medium and allowed to incubate for 24–48 h at 37 °C in 5% CO_2_. To quantify the pseudotube formation, photographs of at least 3 fields per well were taken (Nikon NIS Element, ×10 magnification) and total pseudotube length was quantified using ImageJ software.

### Cell migration assays

Transwell (24 wells, 8 µm pore size, BD Biosciences) cell culture chambers were used. The upper chamber of the inset was seeded with 15 × 10^3^ cells in 100 µl of a serum-free medium (DMEM-F12 for GSC and GDC; EBM-2 for TDEC and HUVEC). The lower chamber was filled with a serum-free medium (DMEM F12 or EBM-2) containing human recombinant-VEGF (50 ng/ml, R&D Systems) or without VEGF as a control. All conditions were duplicated. Cells were allowed to migrate for 5 h at 37 °C in 5% CO_2_. Non-migrated cells were then removed by wiping the upper side of the membranes with a cotton swab. Transmigrated cells were fixed with Paraformaldehyde 4% (ChemCruz) and stained with Hematoxylin (Merck). The number of transmigrated cells was counted by observation with a Nikon ECLIPSE TS100 microscope. The number of migratory cells was determined by subtracting the number of cells that migrate without VEGF from the number of cells that migrate in the presence of VEGF.

### In vivo Matrigel^TM^ Plug Assay

Nude mice were used according to protocol APAFIS#10654-2017071812393169 v2 reviewed and approved by the Institutional Animal Care and Use Committee of the Midi-Pyrénées Region (France).

After 15 days of culture in transdifferentiation medium, 5 × 10^6^ cells were mixed on ice with Growth Factor Reduced Matrigel^**TM**^ (Corning). A subcutaneous injection of this mix was then administered dorsolaterally in 8-week-old female nude mice. For Tie2 kinase inhibition experiments, the Tie2 kinase inhibitor was diluted in water containing 5% Ethanol and 5% Kolliphor® (Sigma-Aldrich). An intraperitoneal injection of the inhibitor (30 mg/kg) or the vehicle was administered twice a day. After 14 days, mice were sacrificed and the plugs were collected, fixed and embedded in paraffin. For histological analysis, the sections were deparaffinized in xylene, then rehydrated in an ethanol series. Staining with Mayer’s Hematoxylin (Merck) and Eosine-Orange G (Merck) was performed prior to dehydration and mounting with a Eukitt solution. The slides were then observed with a Nikon ECLIPSE TS100 microscope. The number of functional blood vessels was determined by counting the number of blood vessels containing red blood cells on photographs of at least 5 random fields per plug. Images were independently coded and then analysed by at least two experienced members of our laboratory in a blinded manner.

### Immunohistochemistry

For the immunohistochemistry of patient GBMs, frozen tumor sections were fixed in 95% ethanol. The slides were then rehydrated and antigen retrieval was performed at 96 °C for 20 min using DAKO buffer pH9 (high pH target retrieval solution, DAKO, Glostrup, Denmark). The primary antibody (see Table S2) was incubated for 30 min at room temperature and then washed in PBS. The secondary step was performed with the EnVision FLEX system (DAKO) for 20 min, and after PBS wash, revealed using a peroxidase label that was visualized with diaminobenzidine (DAKO). Slides were counterstained with hematoxylin, dehydrated, and mounted.

### Immunofluorescence

Immunofluorescence analysis of Matrigel^TM^ plugs was performed on 5 µm paraffin-embedded sections. Sections were deparaffinized in xylene and rehydrated in an ethanol series. Antigen retrieval was performed at 96 °C for 20 min using DAKO buffer − pH9 and washing done with PBS. For staining, a Tyramide SuperBoost^TM^ kit with AlexaFluor^TM^ 488 Tyramide (Invitrogen) was used as follows. Peroxydase activity was blocked using Blocking Buffer for 60 min at room temperature. Then, sections were incubated with primary antibodies for 60 min at room temperature. The primary antibodies used are indicated in Table S2. After washes in PBS, incubation with the poly-HRP-conjugated secondary antibody was performed for 60 min. Sections were then washed three times in PBS and incubated with Alexa Fluor^TM^ 488 tyramide reagent solution. After 5 min, the reaction was stopped with Reaction Stop Reagent and slides were washed with PBS. Cell nuclei were then marked with DAPI (0.1%, Sigma) prior to mounting the slides using DAKO Fluo mounting medium. For vessels counting, images were independently coded and then analysed by at least two experienced members of our laboratory in a blinded manner.

### Microarray Analysis

The Affymetrix chips were standardized by the RMA method (R software version 3.3.2, Bioconductor version 3.4). The differences between the different conditions were tested using an ANOVA (R software version 3.3.2) corrected for multiple tests using the Benjamini & Hochberg method. Sample Enrichment Score [23] were computed using the Autocompare_SES software (available at https://sites.google.com/site/fredsoftwares/products/autocompare_ses) using the “greater” (indicating an enriched gene set) Wilcoxon tests and the GeneOntology C5 [24], Reactome [25] databases. The genes of the Tie2 signaling pathway used in Fig. 4b are ANGPT2, ANGPT4, DOK2, GRB14, GRB2, GRB7, HRAS, KRAS, NRAS, PIK3CA, PIK3CB, PIK3R1, PIK3R2, PTPN11, SHC1, SOS1 and TEK. All data are available at the National Center of Biotechnology Information (NCBI) GEO Database under the series number GSE138236.

### Statistical analysis

**Fig. 2:**
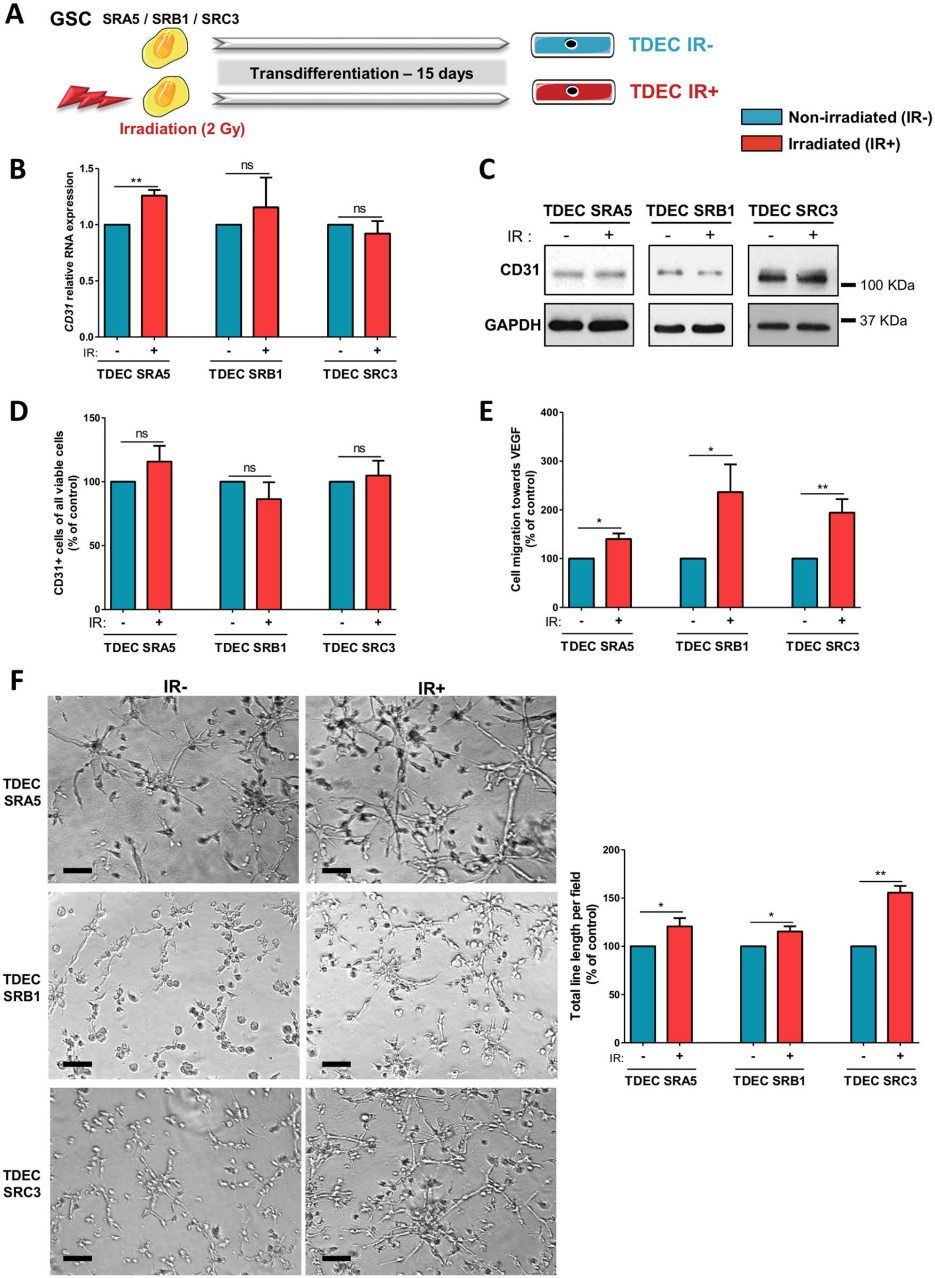
Ionizing radiation potentiates GSC transdifferentiation into TDEC. **a** GSC isolated from tumors of 3 patients (SRA5, SRB1 and SRC3) were or were not irradiated (2 Gy) and were then cultured in EGM-2 for 15 days in order to obtain TDEC IR+ or TDEC IR−. **b** Relative RNA expression of the endothelial marker *CD31* determined by RT-qPCR in TDEC IR− vs. TDEC IR+ from the three different GSC (SRA5, SRB1 and SRC3). The fold inductions are expressed as means ± SEM of at least three independent experiments (normalized to TDEC IR−). **c** Immunoblot of CD31 in TDEC IR− vs. TDEC IR+. Blots are representative of at least 3 independent experiments in the three patients’ GSC lines (SRA5, SRB1, and SRC3). **d** Immunofluorescence analysis by FACS of CD31 protein expression in TDEC IR− vs. TDEC IR+. The graphs represent means SEM of the percentage of CD31 positive cells among all viable cells of at least 3 independent experiments. **e** Cell migration towards VEGF. The graphs represent means ± SEM of the percentage of cells that migrate towards VEGF normalized to TDEC IR−. **f** Pseudotube formation assay. The graphs represent means ± SEM of the total line length per field determined by the quantification of at least 3 fields per well (normalized to TDEC IR−). Scale bars, 100 µm

**Fig. 3:**
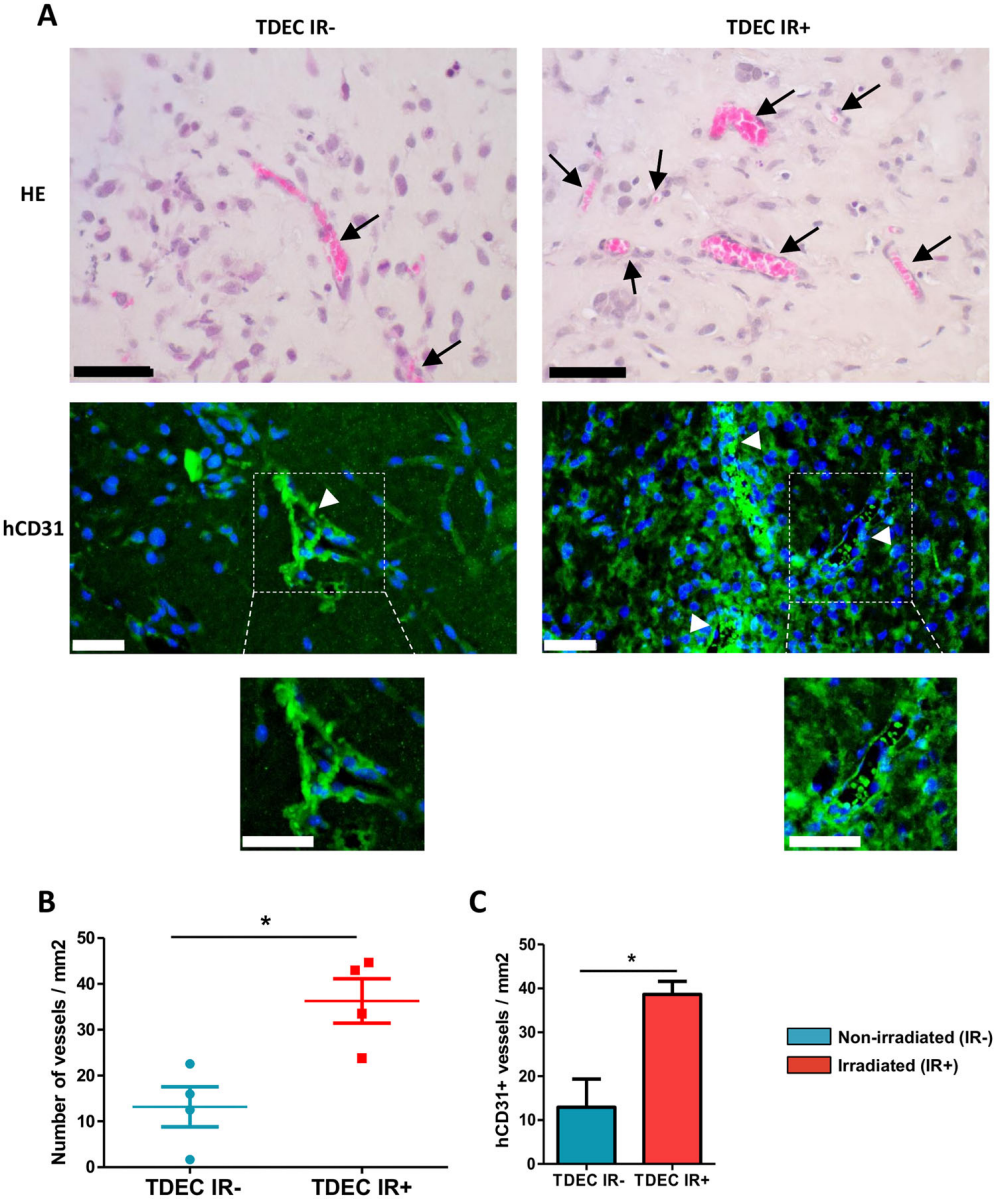
Ionizing radiation potentiates TDEC vessel formation in vivo. **a** Matrigel^TM^ plug assays using TDEC IR− or TDEC IR+ obtained from SRC3 GSC stained by hematoxylin-eosin (upper panel). Immunofluorescence of plugs using a specific anti human-CD31 antibody (lower panel). Arrows indicate functional blood vessels and headed arrows indicate hCD31+ vessels. Scale bars, 50 µm. **b** Quantification of functional blood vessels in Matrigel^TM^ plugs/mm^2^. The number of vessels containing red blood cells per mm^2^ was quantified by histological analysis and was expressed as means ± SEM of 4 mice. **c** Quantification of hCD31+ vessels in Matrigel^TM^ plugs/mm^2^. The number of hCD31 vessels per mm^2^ was quantified and expressed as means ± SEM of 4 mice

**Fig. 4:**
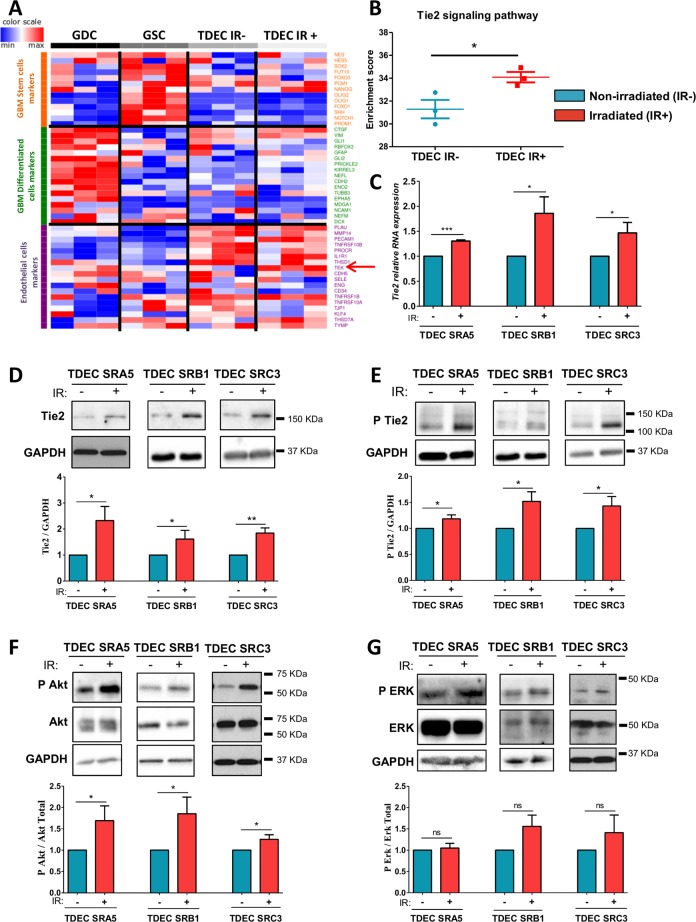
Ionizing radiation increases Tie2 expression and Tie2 signaling pathway activation in TDEC. **a** Transcriptome analysis using Affymetrix® chip was performed on SRC3 GSC, and on GDC, TDEC and TDEC IR+ obtained from SRC3 GSC. Because there are no gene sets for ‘GSC’ or ‘GDC’ in databases like GO, KEGG, Reactome or MSigDB, this gene list was compiled by extensive literature search (Supplementary Table. S3) **b** The enrichment score of Tie2 signaling pathway members in TDEC IR− versus TDEC IR+ was determined from transcriptomic analysis (*n* = 3). The Tie2 signaling pathway members are listed in the “Materials and Methods” section. **c** Relative RNA expression of *Tie2* in TDEC IR− and TDEC IR+ obtained from three different GSC (normalized to TDEC IR− obtained from each GSC). **d**–**g** Immunoblots of Tie2 **d**, P Tie2 **e**, P Akt and Akt **f**, and P ERK and ERK **g** in TDEC IR− and TDEC IR+ obtained from three different GSC. Blots are representative of at least 3 independent experiments. Blots were quantified and the graphs (bottom) show the protein expression ratio normalized to TDEC IR− obtained from each GSC

**Fig. 5:**
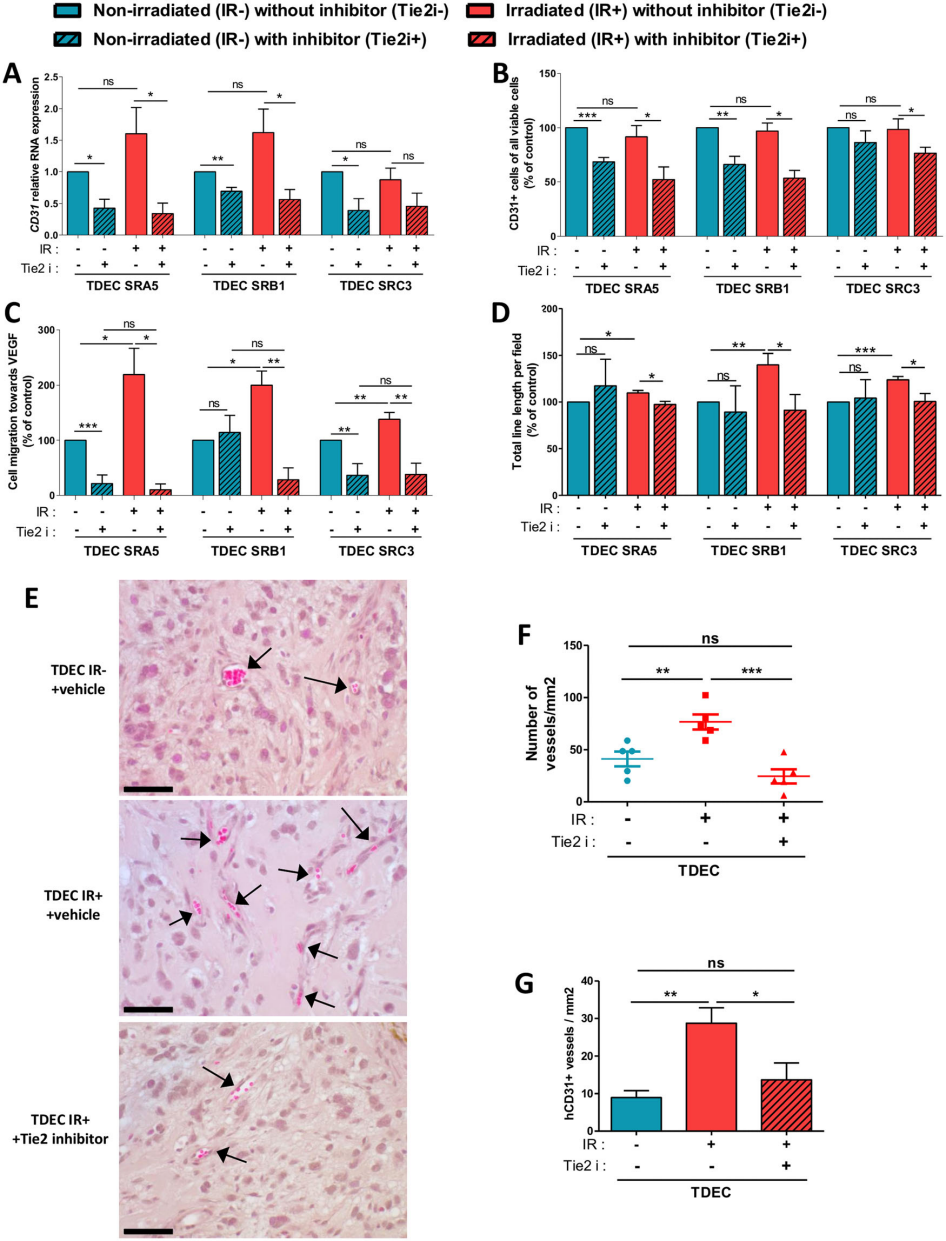
Tie2 kinase inhibitor (Tie2i) decreases the IR-induced effect on TDEC. **a** Relative RNA expression of the endothelial marker *CD31* determined by RT-qPCR in TDEC IR− vs. TDEC IR+ from the three different GSC (SRA5, SRB1 and SRC3) and cultured with or without Tie2i. The fold inductions are expressed as means ± SEM of at least three independent experiments (normalized to TDEC IR− obtained from each GSC and cultured without Tie2i). **b** Flow cytometric analysis of CD31 expression in TDEC IR− and TDEC IR+ obtained from SRA5, SRB1 and SRC3 GSC and cultured with or without Tie2i. The level of CD31 positive cells is expressed as means ± SEM normalized to TDEC IR- obtained from each GSC and cultured without Tie2i. **c** The percentage of cells that migrate towards VEGF normalized to TDEC IR- obtained from each GSC and cultured without Tie2i. **d** Pseudotube formation assay. The graph represents means ± SEM of the total line length per field determined by the quantification of at least 3 fields per well normalized to TDEC IR− without Tie2i obtained from each GSC. **e** Matrigel^TM^ plug assay. Representative hematoxylin-eosin sections of Matrigel^TM^ plugs with TDEC IR− without Tie2i, TDEC IR+ without Tie2i and TDEC IR+ with Tie2i. Black arrows indicate functional blood vessels. Scale bars, 50 µm. **f** Quantification of functional blood vessels in Matrigel^TM^ plugs/mm^2^. The number of vessels/mm^2^ was expressed as means ± SEM of 5 mice for TDEC IR− without Tie2i and TDEC IR+ without Tie2i and of 4 mice for TDEC IR+ with Tie2i. **g** Quantification of hCD31 + vessels in Matrigel^TM^ plugs/mm^2^. The number of hCD31 vessels per mm^2^ was quantified and expressed as means ± SEM of 5 mice for TDEC IR− without Tie2i and TDEC IR+ without Tie2i and of 4 mice for TDEC IR+ with Tie2i

**Fig. 6:**
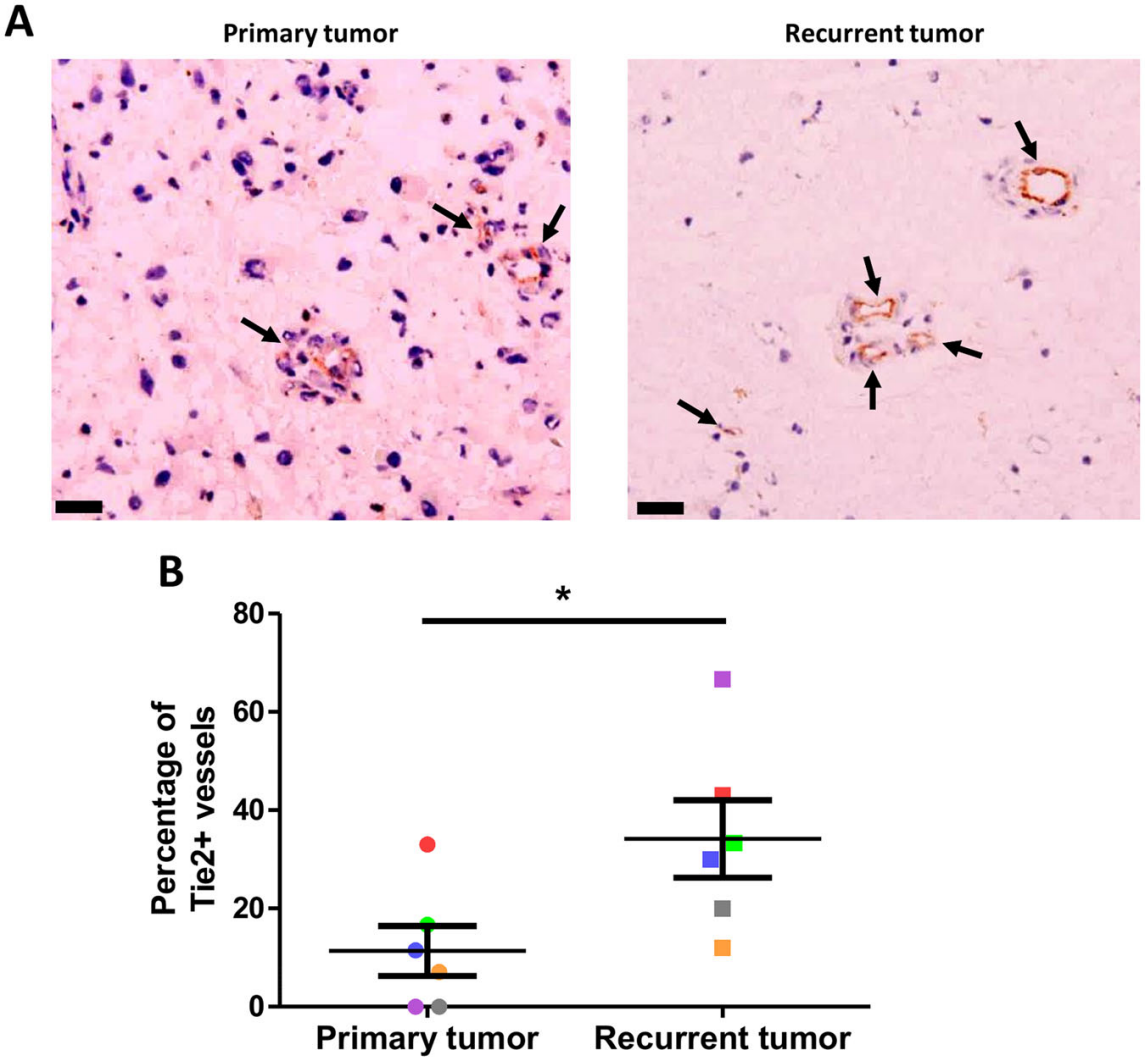
Increase in the percentage of Tie2+ vessels in recurrent GBM. **a** Representative IHC images for Tie2 expression in primary untreated tumors and post-radiation recurrent tumors from matched GBM patients. Arrows indicate functional blood vessels. Scale bars: 50 mm. **b** Percentage of Tie2 positive-vessels in primary untreated tumors and post-radiation recurrent tumors from matched GBM patients (*n* = 6). Each color is associated with one patient. *P < 0.05, by matched-pairs non-parametric Wilcoxon Signed Rank test

For cell culture, each individual experiment was repeated at least three times. Where appropriate, mice were randomized into experimental groups by random number generator. Statistical power was determined from prior studies from our laboratories using similar models/cells [18, 26]. Data were normalized, as appropriate, to means of each experiment with the reference condition set at 1. Experimental data were analysed by unpaired two-tailed *t*-test (for Figs. 1–5) or with matched-pairs non-parametric Wilcoxon Signed Rank test (for Fig. 6). Results are expressed as mean ± SEM. Differences were deemed significant when *P* < 0.05. These analyses were performed using Prism, Version 5.00 (GraphPad Software) or STAT VIEW software package (SAS, Cary, NC).

## Results

### GSC transdifferentiation to TDEC

To study the transdifferentiation of GSC to TDEC, we used three different primary GSC (SRA5, SRB1 and SRC3) established from 3 patients’ surgical GBM samples that are fully characterized in our previous works^17–20^. Therefore, we cultured our GSC in EGM-2 and analyzed the TDEC obtained for endothelial characteristics (Fig. 1). As previously described, TDEC significantly overexpressed RNA and protein of CD31 compared to GDC or GSC (Fig. 1b–d)^11^. In addition, as expected, TDEC lost the expression of stem cells markers (Supp Fig. S1a, b). The expression of TUJ1 was also significantly lower in TDEC SRA5 compared to GDC SRA5^17^. In TDEC SRB1 and TDEC SRC3, TUJ1 expression tends to be lower than in GDC SRB1 and GDC SRC3 but this decrease was not significant. HUVEC also expressed RNA and protein of this marker suggesting that other cells like endothelial cells could also express TUJ1 (Supp Fig. S1a, b). The expression of VEGFR2, the main VEGF receptor, was not significantly different between GSC, GDC and TDEC obtained from SRA5 and SRB1. TDEC obtained from SRC3 GSC expressed significantly more VEGFR2 than SRC3 GSC but the difference was not significant between TDEC and GDC (Supp Fig. S1a). In fact, VEGFR2 is expressed in endothelial cells but many articles indicate that it is expressed in tumor cells and notably in glioma cells^27^.

Furthermore, the expression of smooth muscle cells or pericyte markers (NG2, PDGFRβ, α-SMA, and Calponin) in TDEC from SRA5, SRB1 and SRC3 GSC was lower or not different from the expression of these markers detected in GDC, which confirms that our protocol made it possible to obtain endothelial-like cells and not mural-like cells (Supp Figs. S1a and S2a–c).

However, TDEC obtained from the 3 GSC acquired specific functional characteristics of endothelial cells such as migration to VEGF in the Boyden Chamber, pseudotube formation in Matrigel^TM^ (Fig. 1e, f and Supp Fig. S3) and acLDLuptake (Supp Fig. S4). In fact, we observed a significant increase in TDEC migration towards VEGF compared to GDC or GSC migration of each cell line (Fig. 1e). TDEC were able to form branched structures and pseudotubes in Matrigel^TM^ in contrast to GSC or GDC (Fig. 1f and Supp Fig. S3). The total line length measured for each TDEC cell line was significantly higher in TDEC compared to that in GDC or GSC and was similar to the total line length measured with HUVEC (Fig. 1f). Finally, TDEC obtained from each GSC were able to uptake acetylated low-density lipoprotein (LDL) (Supp. Fig. S4), a specific endothelial capacity^22^.

### Ionizing radiation potentiates TDEC proangiogenic features

To evaluate the impact of IR on TDEC angiogenic features, the 3 different GSC were or were not subjected to a clinical 2-Gy IR and placed 15 days in EGM2 in order to transdifferentiate them into TDEC (Fig. 2a). For the sake of clarity, henceforward, TDEC obtained from non-irradiated GSC will be referred to as TDEC IR− and TDEC obtained from irradiated GSC will be referred to as TDEC IR+. As shown in Fig. 2b, the expression of *CD31* RNA was significantly increased in TDEC IR+ obtained from SRA5 but not from SRB1 and SRC3. Neither CD31 protein expression nor the number of CD31+ cells measured by flow cytometry were significantly increased in TDEC IR+ from SRA5, SRB1 and SRC3 compared to that of TDEC IR− (Fig. 2c, d). Therefore, IR does not increase the number of TDEC obtained from GSC. In parallel, we also confirmed that irradiation of GSC did not affect the RNA or protein expression of stem cell markers (Sox2, Olig2 and NG2) or of differentiated markers (TUJ1) in GSC, in GDC and in TDEC obtained from these 3 GSC (Supp Fig. S5a, b). Moreover, IR did not induce CD31 expression in GSC or GDC of any of the 3 GSC (Supp Fig. S6).

Nonetheless, although IR did not impact the expression of endothelial markers by TDEC, we looked at their specific endothelial abilities such as migration towards VEGF or pseudotube formation in Matrigel^TM^. In fact, we observed a significant increase in TDEC IR+ migration towards VEGF compared to TDEC IR− obtained from SRA5, SRB1 and SRC3 GSC (*P* < 0.05, *P* < 0.05 and *P* < 0.01, respectively) (Fig. 2e) Moreover, IR significantly increased the ability of TDEC IR+ SRA5, SRB1 and SRC3 to form pseudotubes in Matrigel^TM^ compared to TDEC IR− SRA5, SRB1 and SRC3 (*P* < 0.05, *P* < 0.05 and *P* < 0.01, respectively) (Fig. 2f). These effects were not due to an increase in TDEC proliferation because we confirmed that it was not altered by IR (Supp Fig. S7). Overall, the results of functional tests suggest that IR potentiates the proangiogenic abilities of TDEC obtained after transdifferentiation of GSC.

To confirm our results obtained in vitro in in vivo, we used the well-described Matrigel^TM^ plug assay model. Plugs with TDEC IR+ or TDEC IR− were implanted subcutaneously in nude mice and were recovered after 14 days. We then quantified blood vessel ingrowth by counting functional blood vessel (i.e. with red blood cells inside) in each plug (Fig. 3a). As shown in Fig. 3b, plugs with TDEC IR+ contained significantly more functional blood vessels than the Matrigel^TM^ plugs with TDEC IR− (13.18 ± 4.37 vs 36.26 ± 4.83 vessels/mm^2^, *P* < 0.05). To decipher the origin of the vessels observed in each plug, we performed an immunofluorescence assay using a specific anti-human CD31 (hCD31) antibody, which does not stain mouse vessels (Supp Fig. S8). As expected, vessels observed inside the plugs were human vessels labelled by specific anti-hCD31 antibody (Fig. 3a). Therefore, we quantified the number of hCD31+ vessels in all plugs and detected more hCD31+ vessels in plugs with TDEC IR+ compared to plugs with TDEC IR- (Fig. 3c). All these data show that IR potentiates in vitro and in vivo proangiogenic features of TDEC obtained from GSC.

### Involvement of the Tie2 signaling pathway

To decipher the IR-induced transdifferentiation pathways, we performed a transcriptomic analysis which identified that a broad range of specific endothelial genes were upregulated in TDEC obtained from the transdifferentiation of GSC, whereas specific stem cell genes and specific genes of differentiated brain cells were downregulated (Fig. 4a). Interestingly, this transcriptomic analysis allowed us to highlight a clear overexpression of Tie2 in TDEC IR+ compared to TDEC IR−. Similarly, the Sample Enrichment Score analysis of the Tie2 signaling pathway indicated that it was significantly upregulated in TDEC IR+ compared to TDEC IR− (Fig. 4b). To confirm these results, *Tie2* RNA expression was evaluated by RT-qPCR in TDEC IR− and TDEC IR+ obtained from SRA5, SRB1 and SRC3 GSC. As shown in Fig. 4c, *Tie2* RNA was significantly increased in TDEC IR+ obtained from the 3 GSC compared to that in TDEC IR−. Therefore, we looked at the expression of the Tie2 protein and we also found a significant augmentation of its expression in TDEC IR+ compared to TDEC IR− (Fig. 4d). We also examined the activation of Tie2 via phosphorylation. Phosphorylated-Tie2 (P-Tie2)/GAPDH ratio was significantly increased in all three TDEC IR+ compared to TDEC IR− (Fig. 4e). We did not find an increase in the ratio P-Tie2/Tie2, which suggests that the increase in the P-Tie2/GAPDH ratio was due to the increase in Tie2 expression (Supp Fig. S9). Furthermore, IR did not impact RNA expression of *angiopoietin-1* (*ANG1*) and *-2* (*ANG2*), the two ligands of Tie2, in TDEC IR+ compared to TDEC IR− (Supp Fig. S10a, b). In fact, we detected no difference in ANG1 and ANG2 protein levels in supernatants between TDEC IR+ and TDEC IR- (Supp Fig. S10c, d). Tie2 activation may act either via AKT or via ERK pathways. Thus, we then looked at these signaling pathways^28,29^. We identified specific activation of the AKT pathway in TDEC IR+ compared to TDEC IR− (Fig. 4f). We notice no difference in ERK pathway activation between TDEC IR+ and TDEC IR− obtained from the 3 GSC (Fig. 4g).

Therefore, our results showed a significant increase in Tie2 and AKT signaling pathway activation in TDEC IR+, which could explain the effect of IR on the proangiogenic abilities of TDEC IR+.

### Tie2 signaling pathway inhibition decreases IR-induced potentiation of TDEC angiogenic features

To confirm that the Tie2 signaling pathway was involved in IR-induced proangiogenic abilities of TDEC IR+, we decided to inhibit Tie2 activation using a specific Tie2 kinase activity inhibitor (Tie2i)^21^. Using HUVEC, we identified the dose of 2 µM of Tie2i as the optimal concentration required to inhibit the Tie2 signaling pathway (Supp Fig. S11a). Likewise, in the presence of 2 µM of Tie2i, phosphorylation of AKT was clearly decreased in TDEC IR− and in TDEC IR+ (Supp Fig. S11b). Therefore, we inhibited the Tie2 signaling pathway in TDEC IR+ in order to ascertain whether the inhibition of this specific signaling pathway could modify the proangiogenic abilities induced by IR. The inhibition of the Tie2 signaling pathway significantly decreased the expression of *CD31* RNA in all TDEC IR− and TDEC IR+ except in TDEC IR+ SRC3 (Fig. 5a). Similarly, Tie2i induced a significant decrease in the number of CD31+ TDEC obtained from non-irradiated SRA5 and SRB1 GSC, but not from non-irradiated SRC3 GSC (Fig. 5b). However, Tie2i significantly decreased the CD31+ TDEC obtained from all 3 irradiated GSC. As shown in Fig. 5c, Tie2i significantly decreased migration of TDEC IR− SRA5 and SRC3 but not of TDEC IR- SRB1, whereas it significantly decreased migration of TDEC IR+ obtained from all 3 GSC. Interestingly, Tie2i did not influence pseudotube formation of all TDEC IR−, but it significantly decreased pseudotube formation of all 3 TDEC IR+ (Fig. 5d). The proliferation of TDEC IR− and TDEC IR+ was not influenced by Tie2i (Supp Fig. S12). All these results show that, without IR, the Tie2 signaling pathway is involved in transdifferentiation but is not essential for the acquisition of proangiogenic features by TDEC, whereas it plays a crucial role in the IR-induced potentiation of in vitro proangiogenic features of TDEC and particularly in pseudotube formation.

To confirm this important role of the Tie2 signaling pathway, we performed the Matrigel^TM^ plug assay in the presence of Tie2i with TDEC IR+. After subcutaneous implantation of the plugs containing the cells, the mice were injected twice daily with the vehicle or the Tie2i for 14 days^21^. As was previously observed, we found more functional blood vessels in plugs with TDEC IR+ control in comparison to plugs with TDEC IR− control (mean: 76.65 ± 7.34 vs 41.2 ± 7.04 vessels/mm^2^, *P* < 0.01) (Fig. 5e, f). Interestingly, plugs with TDEC IR+ with Tie2i had significantly fewer functional blood vessels than plugs with TDEC IR+ control (24.54 ± 6.81 vs 76.65 ± 7.34 vessels/mm^2^, *P* < 0.001). The number of hCD31+ vessels was also lower in plugs with TDEC IR+ with Tie2i compared to plugs with TDEC IR+ control (Fig. 5g).

All these results suggest that the Tie2 signaling pathway is a key actor of IR-induced potentiation of the proangiogenic features of TDEC.

### Tie2 expression is increased in GBM after treatment

To investigate whether these results were clinically relevant, we collected 6 matched GBM tissues from the initial surgery of untreated tumors and from the second surgery after treatment failure with IR and temozolomide chemotherapy, to evaluate Tie2 expression by immunohistochemistry analysis. Although the number of Tie2+ positive vessels varied among the GBM tissues from the initial surgeries, we observed a significant increase of Tie2+ vessels in recurrent tumors compared to naive tumor sections (Fig. 6a, b). Collectively, Tie2 is upregulated in GBM collected after radiotherapy compared to initial GBM biopsies. This result indicates that Tie2-mediated IR-induced potentiation of proangiogenic features of TDEC can also arise in patients. Therefore, this mechanism could play an important role in tumor recurrence after conventional treatment.

## Discussion

Radiotherapy is a central treatment for GBM. The protocol described by Stupp et al which combines concomitant chemotherapy with temozolomide and radiotherapy (30 daily fractions of 2 Gy) has been the standard of care for patients with GBM for more than a decade since it made it possible to increase the median survival from 12.1 to 14.6 months^3,4^. However, relapse is almost inevitable and mainly occurs at the initial tumor location within the radiation field. The identification of the mechanisms involved in GBM relapse is essential to increase the efficacy of radiotherapy in GBM and thereby increase the overall survival of patients. Here, we report for the first time that 2 Gy irradiation (equivalent to the daily dose used) potentiates proangiogenic features of TDEC in vitro and in vivo through the Tie2 signaling pathway.

It is known that GBM recurrence is preceded by the implementation of a new tumor vasculature^30^. Given the high dose and the wide field of radiation delivered to brain tumors, it is unlikely that any endothelial cells in the tumor could survive^31^. In fact, two articles have shown that irradiation of mice brain tumors induced neovascularization via vasculogenesis^15,16^. Moreover, De Pascalis et al. reported that the percentage of CD31+ cells carrying the same genomic alteration as tumor cells was increased in recurrent tumors compared to primary tumors, which suggests that tumor-derived endothelium plays a role in neo-vascularization^32^. In keeping with this report, we show that an increase in the proangiogenic feature of TDEC obtained from GSC transdifferentiation may also participate in IR-induced neovascularization.

Different authors have shown that GBM cells, and in particular GSC, display significant plasticity^11–13,17,33^. GSC transdifferentiation into endothelial cells is a mechanism which has been described and demonstrated in numerous papers^11–14^. In addition, it was has been shown that GSC can transdifferentiate into other mesenchymal cells such as smooth muscle-like cells or vascular mural-like cells^33,34^. El Hallani et al found that only few CD133+ GSC were able to transdifferentiate into smooth muscle-like cells in vitro which can form tubular structures in Matrigel^TM30^. Scully et al found that GSC could transdifferentiate into vascular mural-like cells to develop vascular mimicry^33^. In these studies, the authors showed that these cells expressed smooth muscle cells or pericyte markers like α-SMA, NG2, or PDGFRβ but expressed no endothelial cell-specific markers such as CD31 or Tie2. In our work, TDEC clearly expressed the endothelial markers CD31 and Tie2 but the expression of smooth muscle cell or pericyte markers (NG2, PDGFRβ, α-SMA and Calponin) is not significantly different from that observed in GDC (Supp Figs. S1 and S2). Moreover, we demonstrated that GDC obtained from the 3 GSC were not able to produce tubular formations when cultured on Matrigel^TM^, which indicates that GDC are not smooth muscle-like cells or vascular mural-like cells^17,33,34^.

To highlight a specific target involved in IR-induced potentiation of proangiogenic features of TDEC, we performed a transcriptomic analysis. In this study, we showed that Tie2 expression as well as the activation of the Tie2 signaling pathway is markedly enhanced in TDEC IR+ compared to TDEC IR−. Tie2 is a tyrosine kinase receptor and is mainly expressed by endothelial cells. Disrupting Tie2 function in mice results in embryonic lethality with defects in embryonic vasculature^35^. Of the three known Tie2 ligands, ANG1 and ANG2 are most often described. In cancer, the Tie2/ANG signaling pathway is clearly involved. For example in GBM, the levels of ANG1, ANG2 and phosphorylated-Tie2 are higher than in normal brain^36,37^. In our study, we clearly demonstrated an increase in Tie2 expression and activation together with an increase in AKT phosphorylation in TDEC IR+, but we showed no difference in ANG1 and ANG2 level, which suggests that the increase in Tie2 activation is mainly due to the increase in its expression. We confirmed that Tie2 activation is involved in IR-induced effect on endothelial features of TDEC by using a specific Tie2 kinase inhibitor in vitro and in vivo. Considering that almost all vessels observed in the plugs were of human origin, it can be assumed that GSC could transdifferentiate into macrophage-like cells since they also express CD31 and Tie2. To our knowledge, the transdifferentiation of GSC into hematopoietic cells such as macrophages has never been described. Furthermore, we detected no increase in macrophage markers (such as CD45, CD68, CD11b or CD14) in TDEC compared to GDC or GSC using the transcriptomic chip data.

Finally, this process of GSC response to radiotherapy, which might allow radioresistant GSC to form new vessels via their transdifferentiation into TDEC, could contribute to the initiation of the tumor recurrence after treatment. In fact, a recent paper using a three dimensional mathematical model, reported that a combinatorial regimen composed of standard treatment currently used (radiotherapy, temozolomide, bevacizumab…) combined with a treatment that would target GSC transdifferentiation into TDEC, could reduce both tumor size and invasiveness and could lead to tumor eradication^38^. Consequently, it would be of great interest to inhibit this particular IR-induced potentiation of the endothelial features of TDEC in order to improve and optimize the treatment of this aggressive brain tumor. It is interesting to note that very recently it was shown that Regorafenib, a pan-tyrosine kinase inhibitor which also targets Tie2, increases the overall survival of patients with recurrent GBM compared to lomustine, which suggests that this efficacy could in part be due to the increase in Tie2 expression after IR that we described^39^. One might put forward that a combination of IR and Regorafenib, which is currently used in the treatment of metastatic colorectal cancer and gastro intestinal stromal tumors, would be an interesting combination for the treatment of primitive GBM.

Overall, our data highlight the existence of a new mechanism of radioresistance in GBM through the transdifferentiation of the surviving GSC after treatment, which leads to the formation of a new tumor vasculature. The IR-induced potentiation of the proangiogenic features of TDEC appeared to be supported by the Tie2 signaling pathway. New therapeutic strategies that combine radiotherapy/temozolomide and a Tie2 signaling pathway inhibitor should be considered for future trials.

## Supplementary information

Suplementary Figures

Supplementary Figures legend

Supplementary Tables

Declaration of contributions to article
