# Supplementary figures and images for: Ionizing radiation induces endothelial transdifferentiation of glioblastoma stem-like cells through the Tie2 signaling pathway
Source: Cell Death Dis. 2019 Oct 28;10(11):816. doi: 10.1038/s41419-019-2055-6 (PMC6817826; doi:10.1038/s41419-019-2055-6)

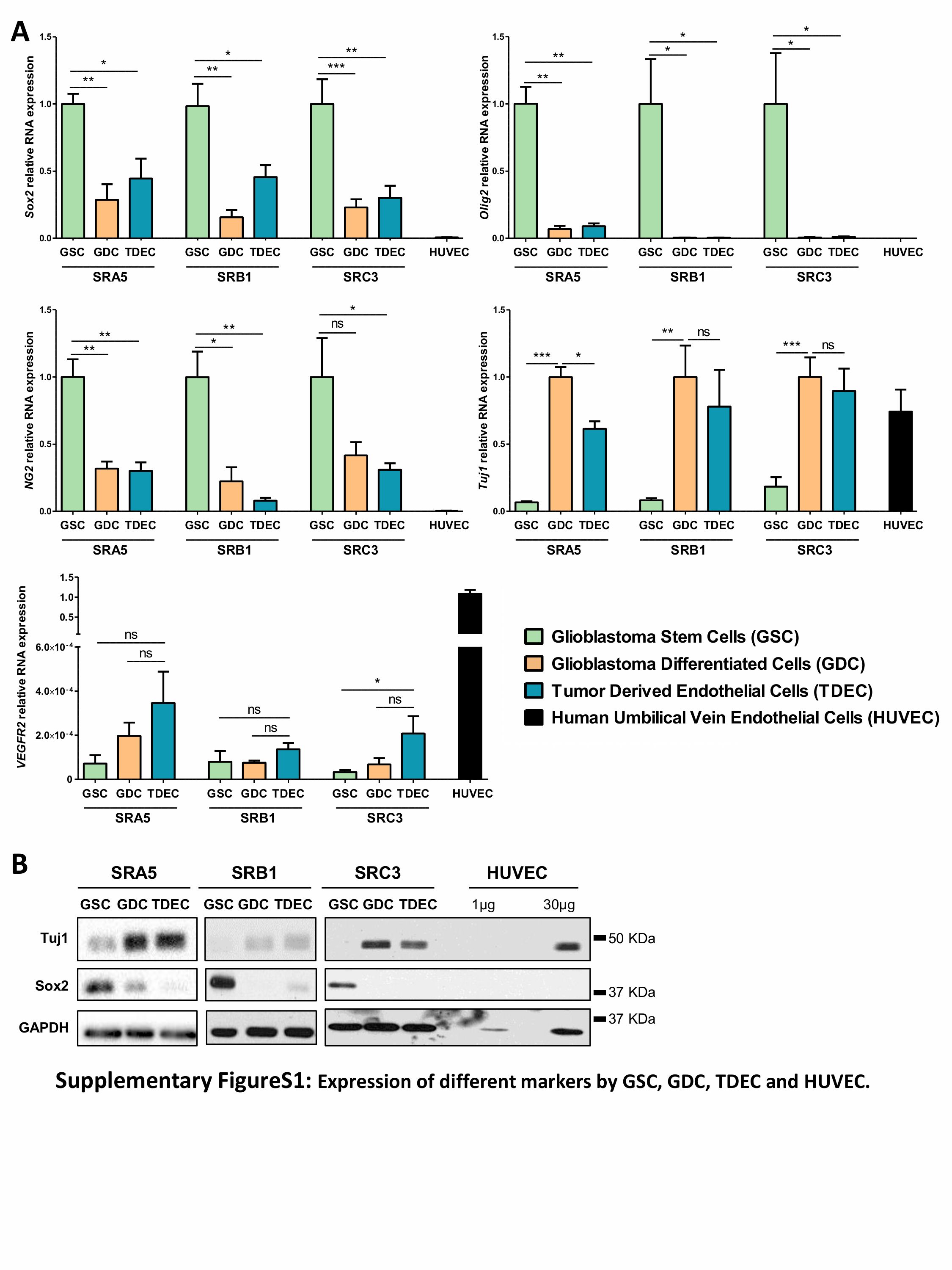

Supplement: Supplementary file 1 — Suplementary Figures [file 41419_2019_2055_MOESM1_ESM.tif]
