# Supplementary material for: Ionizing radiation induces endothelial transdifferentiation of glioblastoma stem-like cells through the Tie2 signaling pathway
Source: Cell Death Dis. 2019 Oct 28;10(11):816. doi: 10.1038/s41419-019-2055-6 (PMC6817826; doi:10.1038/s41419-019-2055-6)
Supplement: Supplementary file 2 — Supplementary Figures legend [file 41419_2019_2055_MOESM2_ESM.doc]

**Supplementary Figures legends**

**Supplementary FigureS1: Expression of different markers by GSC, GDC, TDEC and HUVEC.**

**A.** Relative RNA expression of stem cell markers (Sox2, Olig2 and NG2), differentiation marker Tuj1 and endothelial marker VEGFR2 determined by RT-qPCR in GSC, GDC, TDEC and HUVEC. The fold inductions are expressed as means +/- SEM of at least three independent experiments (normalized to control). ns, not significant; *, *P* < 0.05; **, *P* < 0.01; ***, *P* < 0.001. **B.** Immunoblots of Tuj1 and Sox2 in GSC, GDC, TDEC and HUVEC. Blots are representative of at least 3 independent experiments obtained from the three GSC SRA5, SRB1 and SRC3.

**Supplementary FigureS2: Expression of smooth muscle cell and pericyte markers by GSC, GDC, TDEC and HUVEC.**

**A, B and C:** Relative RNA expression of smooth muscle cell markers (αSMA (A), Calponin (B)) and pericyte marker (PDGFRβ (C)) determined by RT-qPCR in GSC, GDC, TDEC and HUVEC. The fold inductions are expressed as means +/- SEM of at least three independent experiments (normalized to control). ns, not significant; *, *P* < 0.05; **, *P* < 0.01; ***, *P* < 0.001.

**Supplementary FigureS3: Pseudotube formation by GSC, GDC, TDEC and HUVEC.**

Phase contrast pictures showing pseudotube formation of GSC, GDC and TDEC from SRA5, SRB1 and SRC3 and HUVEC. Scale bars : 100µm.

**Supplementary FigureS4: Uptake of acetylated LDL by GDC, TDEC and HUVEC.**

GDC, TDEC or HUVEC were incubated with Di-acLDL (red) for 4 hours. Nuclei were counterstained with DAPI (blue). Pictures are representative of at least three independent experiments. Scale bars: 50µm.

**Supplementary FigureS5: Ionizing radiation does not influence the expression of stem cell and differentiated cell markers.**

**A.** Relative RNA expression of stem cell markers (Sox2, Olig2 and NG2) and the differentiation marker Tuj1 determined by RT-qPCR in GSC, GDC and TDEC. The fold inductions are expressed as means +/- SEM of at least three independent experiments (normalized to control, non-irradiated). ns, not significant. **B.** Immunoblots of Tuj1 and Olig2 in GSC, GDC and TDEC. Blots are representative of at least 3 independent experiments in the three patients’ GSC cell lines (SRA5, SRB1 and SRC3).

**Supplementary FigureS6: Ionizing radiation did not induce CD31 expression in GSC or GDC.**

FACS immunofluorescence analysis of CD31 protein expression in all 3 GSC irradiated or not irradiated and in GDC and TDEC obtained from all 3 GSC irradiated or not irradiated. The graph represents means +/- SEM of the percentage of CD31 positive cells among all viable cells of at least 3 independent experiments. ns, not significant.

**Supplementary FigureS7: Ionizing radiation does not influence TDEC proliferation.**

TDEC were cultured for 2 hours in the presence of WST1 reagent and optical density was then determined in order to assess relative cell proliferation and viability. Relative cell proliferation is expressed as means +/- SEM of at least three independent experiments (normalized to TDEC IR- obtained from each GSC). ns, not significant.

**Supplementary FigureS8: Specificity of CD31 antibodies.**

Immunofluorescence staining for CD31 in the normal human brain (left panels) and the normal mouse brain (right panels) using an anti-human/mouse CD31 (hmCD31) antibody (upper panels) or a specific anti-human CD31 (hCD31) antibody (lower panels). Scale bars : 50 µm**.**

**Supplementary FigureS9: Ionizing radiation does not influence the P Tie2/Tie2 expression ratio.**

At least 3 independent immunoblots of Tie2 and P Tie2 were quantified and the graph shows the P Tie2/Tie2 expression ratio normalized to TDEC IR- obtained from each GSC. ns, not significant.

**Supplementary FigureS10: Expression of ANG1 and ANG2 in TDEC.**

**A and B:** Relative RNA expression of ANG1 (A) and ANG2 (B) determined by RT-qPCR in TDEC obtained from SRB1 GSC and SRC3 GSC. The fold inductions are expressed as means +/- SEM of at least three independent experiments. ns, not significant. **C and D:** Protein level of ANG1 (C) and ANG2 (D) in supernatants of TDEC SRB1 and TDEC SRC3. The means +/- SEM of at least three independent experiments are shown. ns, not significant.

**Supplementary FigureS11: Efficiency of the Tie2 inhibitor.**

**A**. HUVEC were pretreated with different concentrations of Tie2 kinase inhibitor and were then stimulated or not stimulated with angiopoietin 1 (100ng/ml) for 15 min. Phosphorylation of AKT was detected by immunoblotting. **B.** TDEC obtained from SRC3 were pretreated with 2µM of Tie2 kinase inhibitor and were then stimulated or not stimulated with angiopoietin 1 (100ng/ml) for 15 min. Phosphorylation of AKT was detected by immunoblotting and was decreased in the presence of the Tie2 kinase inhibitor.

**Supplementary FigureS12: The Tie2 inhibitor does not influence TDEC proliferation.**

TDEC IR- or TDEC IR+ with or without the Tie2 inhibitor were cultured for 2 hours in the presence of WST1 reagent and the optical density was then determined in order to assess relative cell proliferation. Relative cell proliferation is expressed as means +/- SEM of at least three independent experiments (normalized to TDEC IR- obtained from each GSC). ns, not significant.
