# Supplementary material for: Ionizing radiation induces endothelial transdifferentiation of glioblastoma stem-like cells through the Tie2 signaling pathway
Source: Cell Death Dis. 2019 Oct 28;10(11):816. doi: 10.1038/s41419-019-2055-6 (PMC6817826; doi:10.1038/s41419-019-2055-6)
Supplement: Supplementary file 3 — Supplementary Tables [file 41419_2019_2055_MOESM3_ESM.doc]

**Supplemental data**

**Supplemental Tables**

TableS1: qPCR primers.

| **GENE** | **PRIMER FORWARD (5’ – 3’)** | **PRIMER REVERSE (3’ – 5’)** |
| --- | --- | --- |
| 18S rRNA | TTTCGGAACTGAGGCCATGA | GCAAATGCTTTCGCTCTGGTC |
| CD31 | GCGAGTCATGGCCCGAAGGC | GGTGGTGCTGACATCCGCGA |
| VEGFR2 | TGGGAACCGGAACCTCACTATC | GTCTTTTCCTGGGCACCTTCTATT |
| Tie2 | GCAATGAAGCATGCCACCCTGG | GGTAGCGGCCAGCCAGAAGC |
| Olig2 | CAGAAGCGCTGATGGTCATA | TCGGCAGTTTTGGGTTATTC |
| Sox2 | GCACATGAACGGCTGGAGCAACG | TGCTGCGAGTAGGACATGCTGTAGG |
| NG2 | GCCACGTTGTCAGTCGATG | CCCATAGGGGACCTCTAGGG |
| Tuj1 | GCTCAGGGGCCTTTGGACATCTCTT | TTTTCACACTCCTTCCGCACCACATC |
| ANG1 | CGCCGAAGTCCAGAAAACAG | TCTCAAGTTTTTGCAGCCAC |
| ANG2 | CCCTACGTGTCCAATGCTGT | CCGCTGTTTGGTTCAACAGG |
| PDGFRβ | TCAGCAGCAAGGACACCAT | TGAGGTTGGTCAGTGTGAGC |
| Calponin | GGCCAGCATGGCGAAGACGAAA | TGTGCCCAGCTTGGGGTCGT |
| αSMA | AGCTACCCGCCCAGAAACTA | GTCGCCCACGTAGGAATCTT |

| **PROTEIN** | **PRIMARY ANTIBODY** | **APPLICATION** |
| --- | --- | --- |
| GAPDH (*Human*) | Mouse anti-GAPDH (Calbiochem, CB1001, 1/10000) | Western Blot |
| Olig2 (*Human*) | Rabbit anti-Olig2 (Merck, ab9610, 1/2500) | Western Blot |
| CD31 (*Human*) | Mouse anti-CD31 (Abcam, ab9498, 1/1000) | Western Blot |
| Sox2 (*Human*) | Rabbit anti-Sox2 (Abcam, ab92494, 1/1000) | Western Blot |
| TUJ1 (*Human*) | Mouse anti-TUJ1 (Abcam, ab18207, 1/1000) | Western Blot |
| TIE2 (*Human*) | Mouse anti-Tie2 (Santa-Cruz, sc-293414, 1/200) | Western Blot |
| P TIE2 (*Human*) | Mouse anti-Tie2 (phospho Y992) (Abcam, ab192800, 1/1000) | Western Blot |
| AKT (*Human*) | Rabbit anti-Akt (Cell Signaling, 4691, 1/2000) | Western Blot |
| P AKT (*Human*) | Rabbit anti-phopsho Akt (Ser473) ( Cell Signaling, 4060, 1/1000) | Western Blot |
| ERK (*Human*) | Rabbit anti-p44 MAP Kinase (437S, 1/1000, Cell Signaling) | Western Blot |
| P ERK (*Human*) | Rabbit anti-phospho-p44/42 MAPK (Thr202/Tyr204) (Cell Signaling, 4370, 1/750) | Western Blot |
| CD31 (*Human*) | CD31-APC ( Invitrogen , 17-0319, 1/120) | Flow Cytometry |
| CD31(*Human*) | Rabbit anti-CD31 ( Abcam, ab76533, 1/50) | Immunofluorescence |
| CD31 (*Human/Mouse*) | Rabbit anti-CD31 ( Abcam, ab182981, 1/100) | Immunofluorescence |
| TIE2 (Human) | Mouse anti-Tie2 (Abcam, ab24859, 1/100) | Immunohistochemistry |

TableS2: Antibodies

TableS3: List of genes presented in Fig. 4A that are expressed in GSC, GDC and endothelial cells and supporting references.

| **Abbreviated name** | **Full name** | **Alternative name** | **Reference** |
| --- | --- | --- | --- |
| **GBM stem cells** |  |  |  |
| NES | Nestin |  | Dahan et al. 2014 |
| HES5 | hes family bHLH transcription factor 5 |  | Chen et al. 2013 |
| SOX2 | SRY-box 2 | CD133 | Dahan et al. 2014 |
| FUT10 | fucosyltransferase 10 |  | Kumar et al.2013 |
| FOXO3 | forkhead box O3 |  | Firat et al.2016 |
| PCM1 | pericentriolar material 1 |  | Ge et al. 2010 |
| NANOG | Nanog homeobox | GLI | Dahan et al. 2014 |
| OLIG2 | oligodendrocyte transcription factor 2 |  | Dahan et al. 2014 |
| OLIG1 | oligodendrocyte transcription factor 1 |  | Dahan et al. 2014 |
| FOXO1 | forkhead box O1 |  | Firat et al. 2016 |
| SHH | sonic hedgehog |  | Dahan et al. 2014 |
| NOTCH1 | notch 1 | ARS2 | Dahan et al. 2014 |
| PROM1 | prominin 1 |  | Dahan et al. 2014 |
| **GBM differentiated cells** |  |  |  |
| CCN2 | cellular communication network factor 2 | CTGF, HCS24, IGFBP8, NOV2 | Dahan et al. 2014 |
| VIM | vimentin |  | Jiang et al. 2012 |
| GLI1 | GLI family zinc finger 1 |  | Liao et al. 2016 |
| RBFOX3 | RNA binding fox-1 homolog 3 | FOX3, NeuN | Mullen et al. 1992 |
| GFAP | glial fibrillary acidic protein |  | Dahan et al. 2014 |
| GLI2 | GLI family zinc finger 2 |  | Huang et al. 2018 |
| PRICKLE2 | prickle planar cell polarity protein 2 | EPM5 | Hida et al. 2011 |
| KIRREL3 | kirre like nephrin family adhesion molecule 3 |  | Liu et al. 2015 |
| NEFL | neurofilament light | NFL | Crino et al. 1997 |
| CDH2 | cadherin 2 | NCAD | Ferguson et al. 2012 |
| ENO2 | enolase 2 | NSE | Schmechel et al. 1987 |
| TUBB3 | tubulin beta 3 class III | Tuj1 | Dahan et al. 2014 |
| EPHA5 | EPH receptor A5 | EHK-1 | Miescher et al. 1997 |
| MDGA1 | MAM domain containing glycosylphosphatidylinositol anchor 1 |  | Takeuchi et al. 2007 |
| NCAM1 | neural cell adhesion molecule 1 |  | Weledji et al. 2014 |
| NEFM | neurofilament medium | NFM | Crino et al. 1997 |
| DCX | doublecortin |  | Francis et al. 1999 |
| **Endothelial cells** |  |  |  |
| PLAU | plasminogen activator, urokinase | ATF; QPD; UPA; URK; u-PA; BDPLT5 | Booyse et al. 1984 |
| MMP14 | matrix metallopeptidase 14 | MMP-14; MMP-X1; MT-MMP; MT1MMP; MTMMP1; WNCHRS; MT1-MMP; MT-MMP 1 | Chun et al.2004 |
| PECAM1 | Platelet/endothelial cell adhesion molecule 1 | CD31 | Evrard et al, 2016 |
| TNFRSF10B | TNF receptor superfamily member 10b | CD262, DR5, KILLER, KILLER/DR5, TRAIL-R2, TRAILR2, TRICK2, TRICK2A, TRICK2B, TRICKB, ZTNFR9 | Zhang et al. 2000 |
| PROCR | protein C receptor | CCCA; EPCR; CCD41 | Wei et al. 2018 |
| IL1R1 | interleukin 1 receptor type 1 | P80; IL1R; IL1RA; CD121A; D2S1473; IL-1R-alpha | Wohleb et al. 2014 |
| THSD1 | thrombospondin type 1 domain containing 1 | TMTSP; UNQ3010 | Takayanagi et al. 2006 |
| TEK |  | TIE2; CD202B | Evrard et al, 2016 |
| CDH5 | Cadherin‐5 | VE‐Cadherin; CD144 | Evrard et al, 2016 |
| SELE | E‐selectin | CD62E | Collins et al. 1991 |
| ENG | Endoglin | CD105 | Cheifetz et al. 1992 |
| CD34 | CD34 |  | Evrard et al, 2016 |
| TNFRSF1B | TNF receptor superfamily member 1B | p75; TBPII; TNFBR; TNFR2; CD120b; TNFR1B; TNFR80; TNF-R75; p75TNFR; TNF-R-II | Jasielska et al. 2010 |
| TNFRSF10A | TNF receptor superfamily member 10a | DR4; APO2; CD261; TRAILR1; TRAILR-1 | Zhang et al. 2000 |
| TJP1 | tight junction protein 1 | ZO-1 | Liu et al 2018 |
| KLF4 | Kruppel like factor 4 | EZF; GKLF | Mazzanti et al. 2004 |
| THSD7A | thrombospondin type 1 domain containing 7A |  | Wang et al. 2010 |
| TYMP | thymidine phosphorylase | TP; ECGF; ECGF1; MNGIE; MEDPS1; MTDPS1; PDECGF; hPD-ECGF | Chapouly et al. 2015 |

**References**

~~Ashburner M et al. Gene Ontology: tool for the unification of biology. Nature Genetics. 2000;25:25–9.~~

Booyse FM et al. Isolation and characterization of a urokinase-type plasminogen activator (Mr = 54,000) from cultured human endothelial cells indistinguishable from urinary urokinase. J Biol Chem. 1984 Jun 10;259(11):7198-205.

Chapouly C et al. Astrocytic TYMP and VEGFA drive blood-brain barrier opening in inflammatory central nervous system lesions. Brain. 2015 Jun;138(Pt 6):1548-67.

Cheifetz S. et al. Endoglin is a component of the transforming growth factor‐beta receptor system in human endothelial cells. J Biol Chem, 1992*;* 267, 19027‐19030

Chen X et al. ADAM17 regulates self-renewal and differentiation of U87 glioblastoma stem cells. Neurosci Lett. 2013 Mar 14;537:44-9.

Chun TH et al. MT1-MMP-dependent neovessel formation within the confines of the three-dimensional extracellular matrix. J Cell Biol. 2004 Nov 22;167(4):757-67

Collins, T. et al*.* Structure and chromosomal location of the gene for endothelial‐leukocyte adhesion molecule 1. J Biol Chem, 1991*;*266, 2466‐2473.

Crino PB et al. Internexin, MAP1B, and nestin in cortical dysplasia as markers of developmental maturity. Acta Neuropathol. 1997 Jun;93(6):619-27.

Dahan P, et al. Ionizing radiations sustain glioblastoma cell dedifferentiation to a stem-like phenotype through survivin: possible involvement in radioresistance. Cell Death Dis. 2014 Nov 27;5:e1543. doi: 10.1038/cddis.2014.509.

Evrard SM et al. Endothelial to mesenchymal transition is common in atherosclerotic lesions and is associated with plaque instability. Nat Commun. 2016; 7: 11853

Ferguson TA, Scherer SS. Neuronal cadherin (NCAD) increases sensory neurite formation and outgrowth on astrocytes. Neurosci Lett. 2012 Aug 1;522(2):108-12.

Firat E, Niedermann G. FoxO proteins or loss of functional p53 maintain stemness of glioblastoma stem cells and survival after ionizing radiation plus PI3K/mTOR inhibition. Oncotarget. 2016 Aug 23;7(34):54883-54896.

Francis F et al. Doublecortin is a developmentally regulated, microtubule-associated protein expressed in migrating and differentiating neurons. Neuron. 1999;23:247-56

Ge X et al. Hook3 interacts with PCM1 to regulate pericentriolar material assembly and the timing of neurogenesis. Neuron. 2010 Jan 28;65(2):191-203.

Hida Y et al. Prickle2 is localized in the postsynaptic density and interacts with PSD-95 and NMDA receptors in the brain. J Biochem. 2011 Jun;149(6):693-700.

Huang D et al. GLI2 promotes cell proliferation and migration through transcriptional activation of ARHGEF16 in human glioma cells. J Exp Clin Cancer Res. 2018 Oct 11;37(1):247.

Jasielska M et al. Differential role of tumor necrosis factor (TNF)-alpha receptors in the development of choroidal neovascularization. Invest Ophthalmol Vis Sci. 2010 Aug;51(8):3874-83.

Jiang SX et al. Vimentin participates in microglia activation and neurotoxicity in cerebral ischemia. J Neurochem. 2012 Aug;122(4):764-74.

Kumar A et al. The Lewis X-related α1,3-fucosyltransferase, Fut10, is required for the maintenance of stem cell populations. J Biol Chem. 2013 Oct 4;288(40):28859-68.

Liao Z-Q et al. Glioma-Associated Oncogene Homolog1 (Gli1)-Aquaporin1 pathway promotes glioma cell metastasis. BMB Rep. 2016 Jul 31; 49(7): 394–399.

Liu L et al. Angiotensin II inhibits the protein expression of ZO‑1 in vascular endothelial cells by downregulating VE‑cadherin. Mol Med Rep. 2018 Jul;18(1):429-434.

Liu YF et al. Autism and Intellectual Disability-Associated KIRREL3 Interacts with Neuronal Proteins MAP1B and MYO16 with Potential Roles in Neurodevelopment. PLoS One. 2015 Apr 22;10(4):e0123106.

Mazzanti CM et al. Early genetic mechanisms underlying the inhibitory effects of endostatin and fumagillin on human endothelial cells.Genome Res. 2004 Aug;14(8):1585-93.

Miescher GC et al. Extensive splice variation and localization of the EHK-1 receptor tyrosine kinase in adult human brain and glial tumors. Brain Res Mol Brain Res. 1997 Jun;46(1-2):17-24.

~~Milacic M, et al. Annotating Cancer Variants and Anti-Cancer Therapeutics in Reactome. Cancers. 2012;4:1180–211.~~

Mullen R et al. NeuN, a neuronal specific nuclear protein in vertebrates. Development. 1992;116:201-11

Schmechel DE et al. Localization of neuron-specific enolase (NSE) mRNA in human brain. Neurosci Lett. 1987 May 6;76(2):233-8.

Takayanagi S et al. Genetic marking of hematopoietic stem and endothelial cells: identification of the Tmtsp gene encoding a novel cell surface protein with the thrombospondin-1 domain. Blood. 2006 Jun 1;107(11):4317-25

Takeuchi A et al. Novel IgCAM, MDGA1, expressed in unique cortical area- and layer-specific patterns and transiently by distinct forebrain populations of Cajal-Retzius neurons. Cereb Cortex. 2007 Jul;17(7):1531-41.

~~Tosolini M, et al. Large-scale microarray profiling reveals four stages of immune escape in non-Hodgkin lymphomas. Oncoimmunology. 2016;5.~~

~~Voyta JC, et al. Identification and isolation of endothelial cells based on their increased uptake of acetylated-low density lipoprotein. J Cell Biol. 1984;99:2034–40.~~

Wang CH et al. Thrombospondin type I domain containing 7A (THSD7A) mediates endothelial cell migration and tube formation. J Cell Physiol. 2010 Mar;222(3):685-94.

~~Wang R et al. Glioblastoma stem-like cells give rise to tumour endothelium. Nature. 2010;468:829–33~~

Wei Y et al. Effect of cigarette smoke extract and nicotine on the expression of thrombomodulin and endothelial protein C receptor in cultured human umbilical vein endothelial cells. Mol Med Rep. 2018 Jan;17(1):1724-1730

Weledji EP, Assob JC. The ubiquitous neural cell adhesion molecule (N-CAM). Ann Med Surg (Lond). 2014 Jul 23;3(3):77-81.

Wohleb ES et al. Knockdown of interleukin-1 receptor type-1 on endothelial cells attenuated stress-induced neuroinflammation and prevented anxiety-like behavior. J Neurosci. 2014 Feb 12;34(7):2583-91.

Zhang XD et al. Mechanisms of resistance of normal cells to TRAIL induced apoptosis vary between different cell types. FEBS Lett. 2000 Oct 6;482(3):193-9.
