# Supplementary material for: Ionizing radiation induces endothelial transdifferentiation of glioblastoma stem-like cells through the Tie2 signaling pathway
Source: Cell Death Dis. 2019 Oct 28;10(11):816. doi: 10.1038/s41419-019-2055-6 (PMC6817826; doi:10.1038/s41419-019-2055-6)
Supplement: Supplementary file 4 — Declaration of contributions to article [file 41419_2019_2055_MOESM4_ESM.pdf]

DECLARATION OF CONTRIBUTIONS TO ARTICLE

ADMC

Manuscript Number:	Journal Name:	
CDDIS-19-1900RR	*Cell Death & Differentiation*	(the 'Journal')

Proposed Title of the Contribution:

Ionizing radiation induces endothelial transdifferentiation of glioblastoma stem-like cells through the Tie2 signaling pathway (the 'Contribution)

Author(s):

Pauline Deshors, Christine Toulas, Florent Arnauduc, Laure Malric, Aurore Siegfried, Yvan Nicaise, Anthony Lemarié, Dorian Larrieu, Marie Tosolini, Elizabeth Cohen-Jonathan Moyal, Monique Courtade-Saidi, Solène M. Evrard (the 'Authors')

For all *CDD* articles, each person named as an author in the published version must be able to show he or she has contributed substantially to the article.

Authorship credit should be based on 1) substantial contributions to conception and design, acquisition of data, or analysis and interpretation of data; 2) drafting the article or revising it critically for important intellectual content; and 3) final approval of the version to be published. Authors should meet conditions 1, 2 and 3.

Any person who cannot be shown to have made a substantial contribution to the article cannot be listed as an author in the final version. The name of any person who is deemed to have made a minor contribution can, however, appear in the Acknowledgments section of the article.

Please complete the table below to indicate the contributions of all named authors to the manuscript.

Author Full Name:	Specification of Contribution to the Manuscript:
Pauline Deshors	approved the final version of the manuscriptconceived and designed the experiments;performed experiments;analysed the data;contributed to the writing of the manuscript;
Christine Toulas	conceived and designed the experiments;contributed reagents/materials/analysis tools/technical expertise;analysed the data;contributed to the writing of the manuscript;approved the final version of the manuscript
Florent Arnauduc	conceived and designed the experiments;contributed reagents/materials/analysis tools/technical expertise; performed experiments;contributed to the writing of the manuscript;approved the final version of the manuscript
Laure Malric	contributed reagents/materials/analysis tools/technical expertise;performed experiments;contributed to the writing of the manuscript;approved the final version of the manuscript
Aurore Siegfried	contributed reagents/materials/analysis tools/technical expertise;contributed to the writing of the manuscript;approved the final version of the manuscript
Yvan Nicaise	contributed reagents/materials/analysis tools/technical expertise;contributed to the writing of the manuscript;approved the final version of the manuscript
Anthony Lemarié	conceived and designed the experiments;contributed reagents/materials/analysis tools/technical expertise;performed experiments;contributed to the writing of the manuscript;approved the final version of the manuscript
Dorian Larrieu	contributed reagents/materials/analysis tools/technical expertise;analysed the data;contributed to the writing of the manuscript;approved the final version of the manuscript
Marie Tosolini	contributed reagents/materials/analysis tools/technical expertise;analysed the data;contributed to the writing of the manuscript;approved the final version of the manuscript
Elizabeth Cohen-Jonathan Moyal	contributed to the writing of the manuscript;conceived and designed the experiments;contributed reagents/materials/analysis tools/technical expertise;analysed the data;approved the final version of the manuscript
Monique Courtade-Saidi	conceived and designed the experiments;contributed reagents/materials/analysis tools/technical expertise;performed experiments;analysed the data;contributed to the writing of the manuscript;approved the final version of the manuscript
Solène M. Evrard	conceived and designed the experiments;contributed reagents/materials/analysis tools/technical expertise;performed experiments;analysed the data;contributed to the writing of the manuscript;approved the final version of the manuscript

Please complete the table below to indicate the contributions of all named authors to the figures.

Figure 1:

P.D. and S.E. created and realized the scheme.

Figure 2:

P.D., L.M., A.L. and D.L generated the data with the cells, P.D and S.E. labelled the images, P.D. and S.E. assembled the Figure

Figure 3:

P.D., L.M. and D.L generated the data with the cells, F.A. generated the immunohistochemistry and immunofluorescence data of panel G, P.D and S.E. performed the mouse experiments, F.A. and Y.N. generated the immunofluorescence images of panel G, P.D. and S.E. labelled the images, P.D. and S.E. assembled the Figure, M.C.S, P.D. and S.E. performed the counting for panel H and I

Figure 4:

M.T. analyzed the data from the Affymetrix chip and created the heatmap, P.D. generated the western blot and quantified them, P.D. and S.E. assembled the panels.

Figure 5:

P.D. generated the data with the cells, , P.D and S.E. performed the mouse experiments; F.A. generated the immunohistochemistry and immunofluorescence data of panel E, P.D. and S.E. labelled the images, P.D. and S.E. assembled the Figure, M.C.S, P.D. and S.E. performed the counting for panel F and G

Figure 6:

A.S. identified the GBM patients with recurrence, M.C.S. performed and analyzed the immunohistochemistry, S.E. labelled the figure (panel A) and prepared panel B.

Signed for and on behalf of the Author(s):	Print Name:	Date:
	Solene Evrard	03/10/2019
